# Region-Specific NRF2 Signaling in HIV-Associated Neurocognitive Disorders: A Transcriptomic and Computational Histology Study

**DOI:** 10.3390/genes17020195

**Published:** 2026-02-05

**Authors:** Grazia Scuderi, Serena Spampinato, Michelino Di Rosa, Paolo Fagone, Giuseppe Nunnari

**Affiliations:** 1 Department of Biomedical and Biotechnological Sciences, University of Catania, 95123 Catania, Italy; graziascuderi@hotmail.it (G.S.); mdirosa@unict.it (M.D.R.); 2 Unit of Infectious Diseases, ARNAS “Garibaldi Nesima” Hospital, Department of Clinical and Experimental Medicine, University of Catania, 95122 Catania, Italy; serenaspampinato93@gmail.com (S.S.); giuseppe.nunnari1@unict.it (G.N.)

**Keywords:** neurodegeneration, HIV, HAND, oxidative stress

## Abstract

Background/Objectives. Oxidative stress is a key contributor to HIV-associated neurocognitive disorders (HANDs), yet the regional organization and functional engagement of the NRF2 antioxidant pathway in the human brain remain incompletely defined. This study aimed to characterize NRF2 pathway architecture, baseline brain expression, and disease-associated transcriptional and coexpression remodeling across HAND stages. Methods. The NRF2 signaling network was reconstructed using curated pathway data and protein–protein interaction analysis to identify central hub genes. Baseline expression in the normal human cortex was assessed using the Human Protein Atlas. Transcriptomic profiling of postmortem brain samples from individuals with HAND (GSE35864) was performed using differential expression, hierarchical clustering, and region-specific coexpression analyses across white matter, frontal cortex, and basal ganglia. Results. Low-to-medium baseline expression of NRF2-related genes was observed in the normal cortex. Bulk differential expression revealed minimal NRF2 pathway modulation in the frontal cortex and basal ganglia. On the other hand, white matter exhibited robust NRF2 transcriptional activation specifically in HIV encephalitis (HIVE). Coexpression analysis performed specifically within HAND samples revealed a highly coordinated transcriptional organization of the NRF2 signaling network across all analyzed brain regions. Conclusions. NRF2 signaling in HAND is preserved as a coordinated transcriptional network but is selectively activated in white matter during encephalitic disease, highlighting region- and cell-type-targeted therapeutic opportunities.

## 1. Introduction

HIV-associated neurocognitive disorders (HANDs) remain among the most serious neurological complications of HIV-1 infection, particularly affecting individuals with AIDS under 60 years of age [1,2,3,4]. HAND arises largely from the deleterious effects of HIV-1 proteins on microglial cells, which initiate progressive injury to astrocytes and neurons [5]. Substantial evidence identifies oxidative stress as a central mechanism linking HIV infection to neuronal damage and neurodegeneration [6]. Markers of oxidative DNA damage, such as mitochondrial 8-oxo-2′-deoxyguanosine (8-oxoG), are inversely correlated with grey matter volume in key cognitive regions, including the hippocampus and pallidum, implicating mitochondrial dysfunction in cognitive decline. Increased nuclear 8-oxoG levels coupled with reduced mitochondrial DNA content in the frontal cortex further highlight the role of reactive oxygen species (ROS) in HIV-associated neurological deterioration [7,8].

HIV replication generates unintegrated viral DNA forms, such as linear DNA and 2-LTR circles [9], which activate the cGAS–STING pathway and sustain innate immune activation and chronic inflammation [10]. This inflammatory environment amplifies ROS production and can induce ferroptosis, an iron-dependent, lipid peroxidation-driven form of cell death that compromises neuronal integrity [10]. These processes likely contribute significantly to HAND-associated neuronal injury.

HIV-1 proteins, particularly gp120, Tat, Vpr, Nef, and reverse transcriptase (RT), play pivotal roles in disrupting redox homeostasis. Gp120, Tat, and Vpr cross the blood–brain barrier through redox-sensitive mechanisms involving MMP-2 and MMP-9, allowing their entry into the central nervous system. Once inside, these proteins directly induce excessive ROS generation, overwhelming antioxidant defenses and causing oxidative DNA damage. This damage inhibits DNA repair enzymes such as OGG1, destabilizes mitochondrial DNA, and promotes neuronal apoptosis. Concurrently, ROS-driven lipid peroxidation and ceramide accumulation further disrupt cellular membranes and homeostasis [11,12,13,14].

Indirectly, gp120, Tat, and Vpr activate astrocytes and microglia to release proinflammatory cytokines and chemokines, including TNF-α, IL-6, IL-8, MCP-1, and IL-1β, reinforcing a vicious cycle of inflammation and oxidative stress. Gp120 also alters neuronal excitability through ROS-dependent modulation of potassium currents and enhances NMDA receptor activity, leading to calcium overload and apoptotic cell death [8,15,16,17]. Dopaminergic neurons are particularly vulnerable to gp120-induced oxidative injury, providing a mechanistic basis for the motivational and cognitive symptoms observed in HAND [18,19]. Antioxidant strategies effectively counteract gp120-mediated neurotoxicity in experimental models [20].

Tat further exacerbates oxidative stress by activating NADPH oxidases, spermine oxidase, and mitochondrial dysfunction, leading to mitochondrial DNA leakage and NLRP3 inflammasome activation. This process drives IL-1β production via ROS-dependent NF-κB signaling and promotes neuronal apoptosis through microglia-derived neurotoxic factors [21,22]. Nef modulates NADPH oxidase activity, inducing a biphasic oxidative response that enhances inflammasome activation and chronic inflammation [23,24,25,26]. Vpr induces mitochondrial ROS, membrane depolarization, and apoptosis while simultaneously enhancing HIV transcription through redox-sensitive pathways [27,28,29,30]. RT also contributes to ROS generation, lipid peroxidation, and cellular stress responses [31,32].

Alterations in glutathione levels, antioxidant enzyme activity, lipid peroxidation products, and oxidative stress-related proteins in cerebrospinal fluid extracellular vesicles correlate with disease severity and progression [33,34]. Together, these findings position oxidative stress as a unifying driver of HAND pathogenesis and a compelling target for future diagnostic and therapeutic strategies.

In this context, nuclear factor erythroid 2-related factor 2 (NRF2) represents a particularly compelling regulatory axis. NRF2 is widely recognized as the master transcriptional regulator of inducible antioxidant and cytoprotective responses, orchestrating the expression of genes involved in glutathione biosynthesis, thioredoxin and peroxiredoxin systems, xenobiotic detoxification enzymes, iron and heme metabolism, and redox-sensitive transporters [35,36]. Therefore, NRF2 provides a unifying transcriptional framework capable of coordinating broad and sustained antioxidant programs in response to cellular stress.

NRF2 signaling has been implicated in multiple neurodegenerative and neuroinflammatory disorders, including Alzheimer’s disease, Parkinson’s disease, multiple sclerosis, and stroke, where its activation is associated with neuroprotection and resilience to oxidative injury [37,38]. In the context of HIV infection, the regional and cell-type-specific organization of NRF2 signaling in the human brain during HAND has not been systematically investigated.

This study aimed to characterize the organization, regulation, and cell type-specific activity of the NRF2 signaling pathway in HIV-associated neurocognitive disorders (HANDs) using an integrative bioinformatics approach. The study integrated pathway-guided network reconstruction, region-specific differential gene expression analyses, and intra-group coexpression profiling of publicly available bulk microarray data to capture region-specific heterogeneity in NRF2 pathway regulation associated with HIV-related neuropathology.

## 2. Materials and Methods

### 2.1. Definition of the NRF2 Signaling Pathway

The NRF2 (nuclear factor erythroid 2-related factor 2) signaling pathway was defined using the manually curated NRF2 pathway available in WikiPathways (WP2884) (https://www.wikipathways.org/) (accessed on 20 December 2025). The gene set includes key components involved in redox sensing, antioxidant defense, glutathione biosynthesis and recycling, thioredoxin signaling, heme and iron metabolism, phase I and phase II detoxification enzymes, solute carrier transporters, and xenobiotic metabolism. The curated pathway gene list served as a biologically relevant reference for all downstream analyses. Only genes explicitly annotated within WP2884 were considered NRF2-associated for the purposes of this study.

### 2.2. Protein–Protein Interaction Network Construction

To investigate the structural organization and functional connectivity of the NRF2 pathway, protein–protein interaction (PPI) data were retrieved from the STRING database (v. 12). The NRF2 pathway gene list was uploaded to STRING, and interaction networks were generated by integrating multiple evidence channels, including experimentally validated interactions, curated pathway databases, gene coexpression, gene neighborhood, gene fusion events, and computational predictions. Default STRING confidence thresholds were applied to retain high-confidence functional associations while limiting spurious or low-probability interactions. The resulting PPI network was exported in a compatible format and visualized using Cytoscape (v3.9) for network-based analyses.

Network topological properties were analyzed using Cytoscape’s built-in NetworkAnalyzer tool [39]. Degree centrality, defined as the number of direct connections per node, was calculated for all genes within the NRF2 PPI network. Degree was selected as the primary metric to identify highly connected nodes that potentially act as regulatory or organizational hubs within the pathway. The top 10% genes with the highest degree centrality were classified as hub genes, reflecting their potential importance in coordinating NRF2-dependent signaling and redox responses. These hub genes were prioritized for subsequent expression profiling, correlation analyses, and cell-type-specific investigations.

### 2.3. Assessment of Baseline NRF2 Hub Gene Expression in Normal Human Brain

To establish a physiological reference for NRF2 hub gene expression, baseline protein levels were assessed using data from the Human Protein Atlas (HPA) [40]. Immunohistochemical staining profiles from normal human cerebral cortex samples were examined. Expression levels were categorized qualitatively based on reported protein detectability across neuronal and glial cell types.

### 2.4. Transcriptomic Dataset Acquisition and Analysis

Bulk transcriptomic data were obtained from the Gene Expression Omnibus (GEO) repository under accession number GSE35864 [41]. This dataset comprises postmortem brain samples from control individuals (*n* = 6) and HIV-infected subjects classified as HIV without neurocognitive impairment (*n* = 6), HAND (*n* = 7), or HIV encephalitis (HIVE) (*n* = 5). Tissue samples were derived from three anatomically and functionally distinct brain regions, namely white matter, the frontal cortex, and the basal ganglia, for a total of 72 samples. Autopsies were conducted according to a standardized protocol established by the National NeuroAIDS Tissue Consortium (NNTC), and all collection sites adhered to a centralized neuropathology quality assurance program [42]. Study groups were matched based on sample availability with respect to sex, age, ethnicity, postmortem interval, and other relevant clinical and demographic variables. The demographic and clinical characteristics of the patients from whom the samples were derived have been described in detail by Gelman et al. [41]. The submitter-supplied normalized gene expression matrix was used for the analysis. Pre-processing, background correction, and normalization methods of the raw microarray data are detailed in Gelman et al. [41]. Samples were stratified by brain region and clinical diagnosis prior to downstream analyses to preserve region-specific biological data.

Differential expression analysis was performed using the limma package implemented in the Phantasus web-based application (v. 1.31.1) (https://artyomovlab.wustl.edu/phantasus/) (accessed on 20 December 2025) [43]. For each brain region, linear models were fitted independently to assess transcriptional differences between clinical groups. Multiple testing correction was performed using the Benjamini–Hochberg false discovery rate (FDR) method, with adjusted *p* values < 0.05 considered statistically significant. Analyses were restricted to genes belonging to the predefined NRF2 pathway.

To explore global transcriptional patterns within the NRF2 pathway, hierarchical clustering was performed. Clustering analyses were conducted separately for each brain region to avoid confounding regional effects. Pearson’s correlation metrics and average linkage were applied.

To assess coordinated transcriptional regulation within the NRF2 pathway independently of differential expression, gene–gene correlation analyses were performed exclusively within HAND samples. For each brain region, pairwise Pearson correlation coefficients were calculated among the NRF2 pathway genes. Correlation matrices were generated and visualized to evaluate region-specific coexpression patterns.

### 2.5. Statistical Analysis

Data visualization and figure generation were carried out using GraphPad Prism (version 9). Correlation analyses were performed using the Pearson correlation test after verification of data normality by the Shapiro–Wilk test. Correlation matrices were generated using the SRPlot web platform (https://www.bioinformatics.com.cn/en) (accessed on 20 December 2025). Hierarchical clustering (HC) analyses were performed using the Phantasus platform by applying Euclidean distance with average linkage and z-scoring for row standardization. Differential expression analyses were conducted using the limma algorithm within Phantasus (v1.31.1). One-way analysis of variance (ANOVA), implemented in SPSS (v29.0), was used to assess differences in NRF2 gene expression among disease groups. Unless otherwise specified, statistical significance was defined as an adjusted *p* value of <0.05.

## 3. Results

To investigate the organization, baseline brain expression, and disease-associated transcriptional remodeling of the NRF2 signaling pathway in HIV-associated neurocognitive disorders (HANDs), we applied an integrative analytical framework combining network-based analysis, normal human brain expression profiling, and transcriptomic data derived from HAND brain tissues.

### 3.1. Network-Based Identification of Key NRF2 Pathway Hub Genes

First, the NRF2 signaling pathway was reconstructed using the curated WikiPathways NRF2 pathway (WP2884) (Table S1), which encompasses genes involved in oxidative stress sensing, antioxidant defense, glutathione metabolism, and xenobiotic detoxification (Table S1). The pathway was imported into STRING to generate a protein–protein interaction (PPI) network, integrating experimentally validated and predicted functional associations. The resulting interaction network was subsequently analyzed in Cytoscape to assess topological properties (Figure S1).

Network centrality analysis identified a subset of genes with a high degree of connectivity, indicating a prominent role in maintaining network integrity and information flow. Based on degree centrality, the hub genes within the NRF2 pathway were identified as GPX2, NQO1, GCLC, GSTP1, GPX3, GSR, HMOX1, NFE2L2, GCLM, GSTM1, TXNRD1, TXN, and GSTA1 (Table S2).

### 3.2. Expression of NRF2 Hub Genes in the Normal Human Cortex

**Figure 1:**
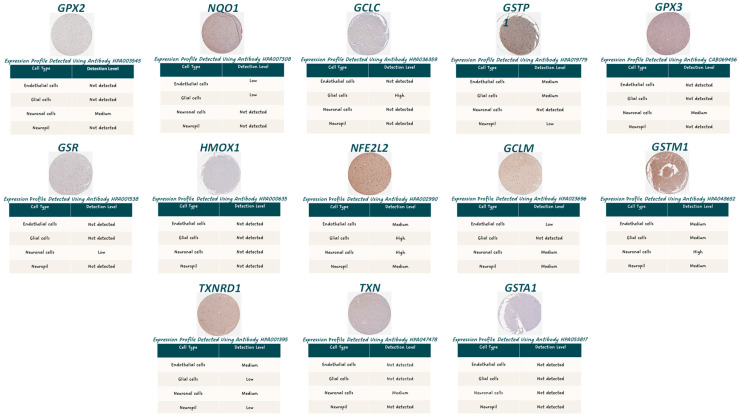
Immunohistochemical staining of NRF2 pathway hub genes in normal human cerebral cortex. Representative immunohistochemistry images from the Human Protein Atlas showing protein expression of NRF2 pathway hub genes in normal human cerebral cortex. Images display antibody-based staining in cortical tissue sections, illustrating cellular localization and relative staining intensity in neuronal and glial cell populations.

To determine whether the identified NRF2 hub genes are physiologically expressed in the human brain, their baseline expression was examined using immunohistochemistry data from the Human Protein Atlas (HPA) (Figure 1).

Expression levels in the normal cortex were mostly low or undetectable. GPX2 and HMOX1 showed no appreciable expression. Similarly, GPX3, which is primarily a secreted glutathione peroxidase expressed in plasma and peripheral tissues, displayed minimal cortical expression. NQO1, GCLC, and GSTP1 exhibited low-level expression signals, but none reached levels indicative of robust constitutive activity in the normal cortex. On the other hand, high expression of GCLC was observed in glial cells, and GSTM1 was expressed at a high level in neuronal cells. Also, NFE2L2 showed high expression in glial and neuronal cells, although both cytoplasmic and nuclear localization could be noted (Figure 1).

### 3.3. Transcriptomic Profiling and Hierarchical Clustering of NRF2 Genes in HAND Brain Regions

**Figure 2:**
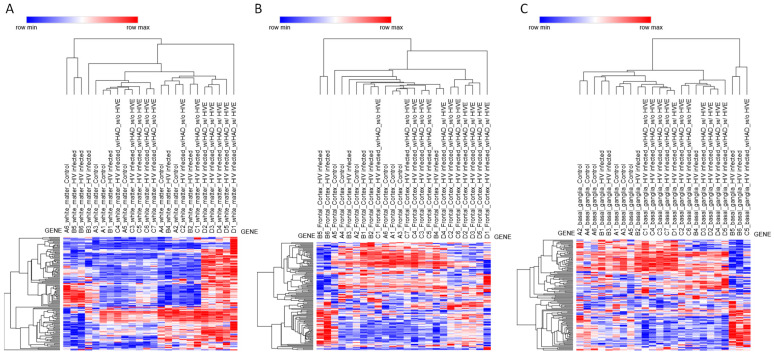
Heatmap and hierarchical clustering of NRF2 pathway gene expression across brain regions. Heatmaps depicting hierarchical clustering of NRF2 pathway gene expression from the GSE35864 transcriptomic dataset. Samples are shown separately for (**A**) white matter, (**B**) frontal cortex, and (**C**) basal ganglia. Rows correspond to NRF2 pathway genes, and columns represent individual brain samples. Hierarchical clustering (HC) analyses were performed using the Phantasus platform by applying Euclidean distance with average linkage and z-scoring for row standardization.

To explore disease-associated changes in NRF2 signaling, transcriptomic data from the GSE35864 dataset were analyzed (Figure 2 and Figure S3, Table S3). This dataset includes postmortem brain samples from individuals with HAND, spanning three anatomically and functionally distinct regions: white matter, frontal cortex, and basal ganglia. Differential expression analysis was performed using the limma algorithm (Tables S4–S6), followed by hierarchical clustering based on NRF2 pathway gene expression. This analysis revealed marked regional differences in NRF2-associated transcriptional modulation, with the most pronounced effects observed in white matter and minimal changes detected in the basal ganglia and frontal cortex.

White matter displayed a distinct and robust transcriptional response associated specifically with HIVE (Figure 2A). No significant differences in NRF2 pathway gene expression were observed in white matter for HIV versus control or HAND versus HIV comparisons, indicating that early or moderate neurocognitive impairment is not associated with detectable NRF2-related transcriptional changes in this compartment. However, comparison of HIVE versus HIV revealed extensive differential expression of NRF2-related genes, with numerous transcripts reaching statistical significance after multiple testing correction (adjusted *p* < 0.05). The genes modulated in white matter during HIVE included MAFG and *KEAP1*, alongside canonical antioxidant and detoxification genes, including *NQO1*, *GSR*, *GCLM*, *G6PD*, *TXNRD1*, *SRXN1*, *BLVRB*, *GSTM4*, *MGST2*, and *MGST3*, as well as several phase I and phase II metabolic enzymes, including *CES2*, *CES3*, *CYP2A6*, *CBR3*, *ADH7*, and *ALDH3A1*. Also, a statistically significant modulation of solute carrier and ABC transporter genes, including multiple members of the *SLC2*, *SLC5*, *SLC6*, and *SLC39* families, as well as *ABCC2*, *ABCC3*, and *ABCC5*, was observed. Growth factor-related genes and stress-responsive signaling mediators, such as *HGF*, *HBEGF*, *EPHA2*, *NRG1*, *EGR1*, and *RXRA*, were also significantly altered (Figure 2A).

By contrast, no statistically significant modulation of NRF2 pathway genes was detected in the frontal cortex for any of the comparisons analyzed. The absence of differentially expressed genes in HIV versus control, HAND versus HIV, and HIVE versus HIV contrasts suggests that, at the transcriptomic level, NRF2-related pathways remain largely unaltered in the frontal cortex, despite the presence of neurocognitive impairment or encephalitic pathology (Figure 2B).

In the basal ganglia, limma analysis did not identify significant differential expression of NRF2-related genes in any of the tested contrasts, including HIV versus control, HAND versus HIV, and HIVE versus HIV. The only exception was the zinc transporter gene SLC39A12, which was modestly upregulated in HIVE compared with HIV (adjusted *p* = 0.041). Aside from this isolated change, the NRF2 pathway appeared transcriptionally stable in the basal ganglia across disease stages, indicating limited engagement of NRF2-dependent stress responses in this region (Figure 2C).

### 3.4. Region-Specific Coexpression Patterns

**Figure 3:**
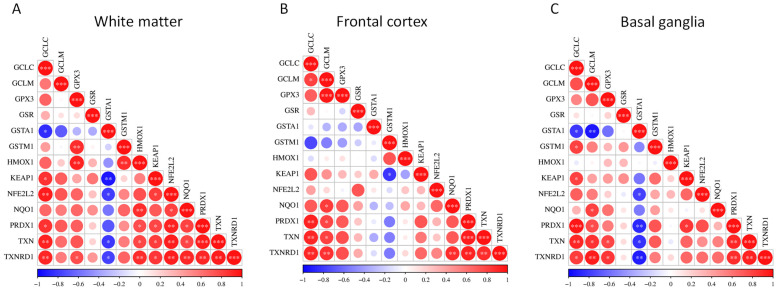
Pairwise correlation matrices of NRF2 pathway hub genes in distinct brain regions. Correlation heatmaps showing pairwise gene–gene expression correlations among NRF2 pathway hub genes using transcriptomic data from the GSE35864 dataset. Correlations were determined within the HAND group only. Separate panels display correlations in (**A**) white matter, (**B**) frontal cortex, and (**C**) basal ganglia. Color gradients represent the strength and direction of correlations between individual gene pairs. * *p* < 0.05; ** *p* < 0.01; *** *p* < 0.001.

Pairwise gene–gene correlations for the hub genes within the NRF2 pathway across the three brain regions were analyzed, interrogating the GSE35864 dataset (Figure 3) (Tables S7–S9).

In white matter, the correlation matrix is characterized by positive associations among the majority of NRF2 pathway hubs. Significant correlations are observed within core antioxidant modules, particularly among *GCLC*, *GCLM*, *GPX3*, *GSR*, *NQO1*, *PRDX1*, *TXN*, and *TXNRD1*, indicating coordinated transcriptional regulation of glutathione synthesis, redox buffering, and peroxide detoxification. *NFE2L2* shows positive correlations with multiple downstream genes, including *NQO1*, *PRDX1*, *TXN*, and *TXNRD1*, while *KEAP1* also exhibits positive associations with several pathway components. In contrast, GST family members display more variable behavior in white matter, with *GSTA1* showing negative correlations with several core NRF2-associated genes, suggesting partial decoupling of specific detoxification enzymes from the broader antioxidant network (Figure 3A).

In the frontal cortex, overall correlation patterns remain largely concordant with those observed in white matter, with extensive positive co-expression among redox-regulatory genes. The strongest correlations again cluster around the thioredoxin system and glutathione-related genes, including *TXN*, *TXNRD1*, *PRDX1*, *GCLC*, and *GCLM*. *HMOX1* demonstrates moderate to strong positive correlations with multiple NRF2-responsive genes. As in white matter, *GSTA1* and, to a lesser extent, *GSTM1* show weaker or negative correlations with several pathway members, indicating region-specific heterogeneity within the glutathione S-transferase subnetwork (Figure 3B).

In the basal ganglia, statistically significant positive correlations were observed among most NRF2 pathway hubs, including *GCLC*, *GCLM*, *GPX3*, *NQO1*, *PRDX1*, *TXN*, and *TXNRD1*. *NFE2L2* and *KEAP1* both showed positive correlations with numerous downstream targets. Notably, negative correlations involving *GSTA1* are more pronounced in the basal ganglia than in the other regions, with strong inverse correlations observed with multiple core redox genes, whereas *GSTM1* displays predominantly positive correlations (Figure 3C).

An exploratory cell-type deconvolution analysis [44] was performed to estimate broad cellular composition across brain regions and clinical groups (Supplementary Figure S3). In white matter, a reduction in inferred oligodendrocyte signatures was observed in HIVE, while frontal cortex samples showed shifts in astrocyte, excitatory neuron, and microglial signatures. No significant compositional differences were detected in the basal ganglia. High-resolution imputation suggested potential alterations in inferred NRF2 hub gene expression in excitatory neurons and oligodendrocytes in HIVE, whereas other major cell classes showed largely stable patterns. Given the use of coarse cell categories and non-region-matched reference signatures, these findings should be interpreted with caution and are presented as hypothesis-generating.

## 4. Discussion

In this study, we provide an integrated network-based and transcriptomic analysis of the NRF2 signaling pathway [45] in the context of HIV-associated neurocognitive disorders [46], with particular emphasis on region- and cell-type-specific modulation in the setting of HIV encephalitis. Although oxidative stress is widely recognized as a central driver of HAND pathogenesis [7,8], the organization, baseline activity, and disease-associated remodeling of the NRF2 antioxidant network in the HAND human brain have remained incompletely defined. Our findings reveal a striking dissociation between the established importance of oxidative stress in HAND and the relatively restricted engagement of NRF2-dependent transcriptional programs at the tissue and cellular levels, with robust activation confined largely to white matter and to specific cellular populations during encephalitic disease.

Network reconstruction of the NRF2 pathway identified GPX2, NQO1, GCLC, GSTP1, GPX3, GSR, HMOX1, NFE2L2, GCLM, GSTM1, TXNRD1, TXN, and GSTA1 [47] as highly connected hub genes, highlighting their central role in coordinating antioxidant defense, glutathione metabolism, and detoxification. Notably, the interrogation of normal human cortical expression data from HPA revealed uniformly low baseline expression of these hubs in the healthy brain. This observation suggests that canonical NRF2-driven detoxification pathways are not constitutively active in cortical tissue under physiological conditions, consistent with the concept that the brain operates under a tightly regulated redox environment and may rely on inducible, rather than basal, NRF2 activation in response to stress. This low baseline expression also provides an important contextual framework for interpreting disease-associated changes, as even modest transcriptional induction may represent biologically meaningful activation.

Although HPA data clearly indicate that, under physiological conditions, the NRF2 pathway is largely inactive, we recognize that HPA has limitations, including incomplete coverage and reduced sensitivity for low-abundance or inducible proteins, which prevent its use as a quantitative baseline for RNA-based analyses. Accordingly, we interpret these observations as contextual, orthogonal evidence supporting the lack of NRF2 activation in the healthy human cortex, rather than as definitive measures of protein abundance or activity. Integrating HPA data with transcriptomic analyses provides a valuable framework to contrast basal versus disease-associated NRF2 engagement while acknowledging that functional validation in experimental models remains necessary.

Contrary to expectations based on the extensive literature implicating oxidative stress in HAND, bulk transcriptomic analysis revealed minimal NRF2 pathway modulation in the frontal cortex and basal ganglia across HIV infection, HAND, and HIVE. In the basal ganglia, the near-complete absence of differential expression, with the exception of modest SLC39A12 upregulation, indicates that NRF2-related transcriptional responses are not broadly engaged in this region despite its known vulnerability in HIV-related neurological disease. Similarly, the lack of detectable NRF2-related transcriptional changes in the frontal cortex suggests that oxidative injury in this region may occur through mechanisms independent of canonical NRF2 transcriptional activation, such as post-transcriptional regulation, impaired NRF2 nuclear translocation, or overwhelming oxidative burden that exceeds compensatory capacity.

In marked contrast, white matter exhibited a robust and highly specific NRF2 transcriptional response associated exclusively with HIVE. The absence of significant changes in HIV versus control or HAND versus HIV comparisons indicates that NRF2 pathway engagement is not a feature of early infection or neurocognitive impairment alone, but rather is tightly linked to encephalitic pathology. The broad induction of NRF2-related genes in white matter, encompassing glutathione metabolism, thioredoxin signaling, xenobiotic detoxification enzymes, transporters, and redox-sensitive transcriptional regulators, suggests a coordinated stress response triggered by severe inflammatory and oxidative conditions characteristic of HIVE. This regional specificity likely reflects the unique vulnerability of white matter to oxidative damage, lipid peroxidation, and metabolic stress, particularly in the context of myelin-rich environments.

Importantly, pairwise correlation analysis of NRF2 pathway genes was performed exclusively within HAND samples, allowing assessment of coordinated transcriptional regulation independent of differential expression across disease groups. Within this context, strong positive correlations among core antioxidant and redox-regulatory genes were observed across all three brain regions. These findings indicate that, even in regions where NRF2 pathway genes are not differentially expressed relative to other clinical groups, NRF2-associated genes remain tightly co-regulated within HAND samples. This coordinated expression suggests preservation of an intact NRF2 transcriptional network, potentially poised for activation, rather than transcriptional disintegration or pathway silencing.

Notably, white matter HAND samples exhibited particularly strong correlations among genes involved in glutathione synthesis, peroxide detoxification, and thioredoxin signaling, consistent with a highly integrated redox regulatory network. Similar, albeit slightly less pronounced, patterns were observed in the frontal cortex and basal ganglia. The presence of strong coexpression in regions lacking overt differential expression reinforces the notion that NRF2 signaling may be regulated primarily at the level of activation dynamics or stress thresholds, rather than through sustained changes in baseline transcript abundance. In addition, cell-type deconvolution analysis suggests that changes in NRF2-related transcriptional activity occur against a backdrop of relatively stable cellular composition in most regions. In white matter, the selective reduction in oligodendrocyte fractions in HIVE, in the absence of significant changes in other cell populations, is consistent with oligodendrocyte vulnerability to oxidative stress and inflammatory injury. Importantly, this compositional shift parallels the robust induction of NRF2-related genes in white matter, suggesting that oligodendrocyte dysfunction and loss may act as both a driver and a consequence of oxidative stress in encephalitic disease.

Pharmacological NRF2 activators, including electrophilic compounds such as dimethyl fumarate (DMF), sulforaphane, and bardoxolone methyl, have demonstrated neuroprotective effects in preclinical models of neurodegeneration by reducing oxidative damage, attenuating neuroinflammation, and preserving mitochondrial function [37]. Notably, DMF has been successfully translated into clinical practice for multiple sclerosis, providing proof-of-concept that pharmacological modulation of NRF2 signaling is both feasible and effective in human neuroinflammatory disease [48,49].

In parallel, antioxidant-based interventions, including glutathione precursors, N-acetylcysteine, and dietary polyphenols, have been evaluated in both preclinical models and limited clinical studies for their ability to mitigate oxidative stress [50]. In the context of HIV infection, however, clinical outcomes have been inconsistent, likely reflecting the complexity of redox regulation in the central nervous system and the absence of pathway-specific therapeutic targeting [50].

Importantly, none of the currently available therapeutic strategies have been optimized to account for region- or cell-type-specific regulation of NRF2 signaling, particularly in HAND. Most existing approaches rely on systemic activation of antioxidant pathways and do not consider the pronounced regional heterogeneity and cellular specialization of the human brain. These limitations may contribute to variable therapeutic efficacy and underscore the need for more precise, biologically informed strategies aimed at selectively modulating NRF2 signaling within vulnerable brain regions or specific brain cell populations.

The present study has some limitations that should be mentioned. First, this study relies on the use of postmortem brain tissue data, which inherently provide a static snapshot of gene expression at end-stage disease. Such analyses cannot capture dynamic pathway activation, transient signaling events, or the temporal progression of molecular responses. This limitation is particularly relevant for NRF2 signaling, which is heavily regulated at post-transcriptional and post-translational levels, including protein stabilization, nuclear translocation, and KEAP1-mediated degradation. Consequently, the absence of differential expression in certain regions does not necessarily indicate a lack of oxidative stress or NRF2 involvement, but may instead reflect transient activation, exhaustion of compensatory responses, or regulation occurring upstream of transcription. Moreover, postmortem tissue represents the cumulative outcome of chronic disease processes rather than early pathogenic events. Therefore, our findings primarily reflect end-stage adaptive or maladaptive NRF2 engagement in HAND rather than real-time signaling dynamics. Despite this limitation, postmortem human brain tissue remains an invaluable resource for investigating region- and cell-type-specific molecular organization in HAND, and the integration of network analysis, coexpression profiling, and computational histology allows us to extract meaningful insights into pathway architecture that cannot be obtained from experimental models alone.

Another notable limitation of our study is the reliance on only five broad cell-type categories, namely astrocytes, excitatory neurons, inhibitory neurons, microglia, and oligodendrocytes, for transcriptomic deconvolution. This coarse categorization is particularly limiting for region-specific analyses, such as in the basal ganglia, where medium spiny neurons constitute the dominant and transcriptionally distinct neuronal population. As a result, the deconvolution results may be difficult to interpret and could reflect transcriptional activation within cell types rather than genuine changes in cell-type composition. Consequently, conclusions drawn regarding cell-type-specific contributions should be considered preliminary and interpreted with caution. Future analyses would benefit from leveraging regionally relevant single-cell RNA-seq reference signatures, as provided by recent human brain atlases, to achieve higher-resolution deconvolution and more accurately capture the complexity of cellular heterogeneity. Despite this limitation, our approach provides a coarse, yet informative, overview of major cell-type contributions to transcriptomic patterns across brain regions.

## 5. Conclusions

Collectively, our results support a model in which oxidative stress in HAND is not uniformly counterbalanced by NRF2 activation across brain regions or cell types. Instead, NRF2-dependent transcriptional responses are selectively engaged in white matter and, apparently, within specific vulnerable populations during encephalitic disease. This restricted activation may contribute to the progressive nature of HAND, as regions and cell types unable to mount adequate NRF2 responses remain susceptible to cumulative oxidative damage.

These findings have important implications for therapeutic strategies targeting oxidative stress in HAND. Broad antioxidant approaches may fail to achieve efficacy if NRF2 activation is regionally constrained or cell-type-specific. Instead, strategies aimed at enhancing NRF2 signaling, or at preventing ferroptotic and mitochondrial damage in white matter, may offer greater neuroprotective potential. Moreover, the selective engagement of NRF2 pathways in HIVE highlights the need to consider encephalitic status when evaluating biomarkers or interventions targeting oxidative stress in HIV-associated neurological disease.

## Data Availability

All data used are publicly available under accession GSE35864.

## Supplementary Materials

The following supporting information can be downloaded at https://www.mdpi.com/article/10.3390/genes17020195/s1: Figure S1: Nrf2 string network; Figure S2: complete Hierarchical clustering; Figure S3: Cell-type deconvolution and cell type–specific expression of NRF2 pathway hub genes (Com-putational Histology); Table S1: Nrf2 pathway genes; Table S2: Nrf2 network analysis; Table S3: Gene expression matrix for Nrf2-related genes retrieved from GSE35864; Table S4: Limma differential expression analysis for basal ganglia samples; Table S5: Limma differential expression analysis for frontal cortex samples; Table S6: Limma differential expression analysis for white matter samples; Table S7: Correlation analysis for white matter HAND samples; Table S8: Correlation analysis for frontal cortex HAND samples; Table S9: Correlation analysis for basal ganglia HAND samples.
